# Dual frequency sound absorption with an array of shunt loudspeakers

**DOI:** 10.1038/s41598-020-67810-z

**Published:** 2020-07-02

**Authors:** Pengju Zhang, Chaonan Cong, Jiancheng Tao, Xiaojun Qiu

**Affiliations:** 1 Key Laboratory of Modern Acoustics and Institute of Acoustics, Nanjing University, Nanjing, China; 2 Department of Physics, China Jiliang University, Hangzhou, China; 3 Centre for Audio, Acoustics and Vibration, Faculty of Engineering and IT, University of Technology Sydney, Sydney, Australia

**Keywords:** Acoustics, Electrical and electronic engineering

## Abstract

Transformer noise is dominated by low frequency components, which are hard to be controlled with traditional noise control approaches. The shunt loudspeaker consisting of a closed-box loudspeaker and a shunt circuit has been proposed as an effective sound absorber by storing and dissipating the electrical energy converted from the incident sound. In this paper, an array of shunt loudspeakers is proposed to control the 100 Hz and 200 Hz components of transformer noise. The prototype under tests has a thickness of 11.8 cm, which is only 1/28 of the wavelength of 100 Hz. The sound absorption performance of the array under random incidence is analyzed with the parallel impedance method, and the arrangement of array elements is optimized. The test results in a reverberation room show that the proposed array has sound absorption coefficients of 1.04 and 0.93 at 100 Hz and 200 Hz, respectively, which provides potential of applying this type of thin absorbers for low-frequency sound control.

## Introduction

Effective absorption of the low frequency sound is a challenge in noise control and architectural acoustics. Traditional absorbers usually require large back cavity or thick depth for low frequency sound absorption^1–5^. Acoustic metamaterials have been studied intensively due to their subwavelength size^6–20^. For example, thickness of metamaterial absorbers can be significantly reduced with space coiling or folding^6–9^; however, the absorption performance is hard to be tuned after they are manufactured^8,10^. Membrane based metamaterials^11–13^ and resonance coupling metamaterials^14–18^ require specific elastic properties or Q-factors to attain optimal absorption performance, but they are difficult to be manufactured for large-scale applications. Active noise control technology^21–23^ has problems of the system cost, complexity and robustness.

A typical shunt loudspeaker (SL) is composed of a closed-box loudspeaker with a shunt circuit connected to its terminals. Its absorption performance can be adjusted by tuning the shunt circuit. The SL was firstly proposed for the sound field control in a duct^24^ and its working mechanism was proven to be equal to that of a feedback active control system^25^. Negative impedance converters were employed to adjust the SLs flexibly by tuning the negative resistances, inductances or capacitances implemented in the shunt circuit^26^, and micro-perforated panels were combined to extend the absorption frequency band of the SLs^27,28^. Dual-resonance and multi-resonance absorbing SLs were also investigated for multi-tonal noise absorption^29–31^, where the normal absorption coefficients of the designed SL are larger than 0.9 at 100 Hz and its harmonic frequencies. These results demonstrate that a single SL can be an effective and adjustable sound absorber. For practical applications, multiple SLs have to be used. Although several SLs have been used for the room mode equalizations^32,33^, the sound absorption performance of SL arrays under the random incidence is unknown, which is reported in this paper.

## Results

**Figure 1:**
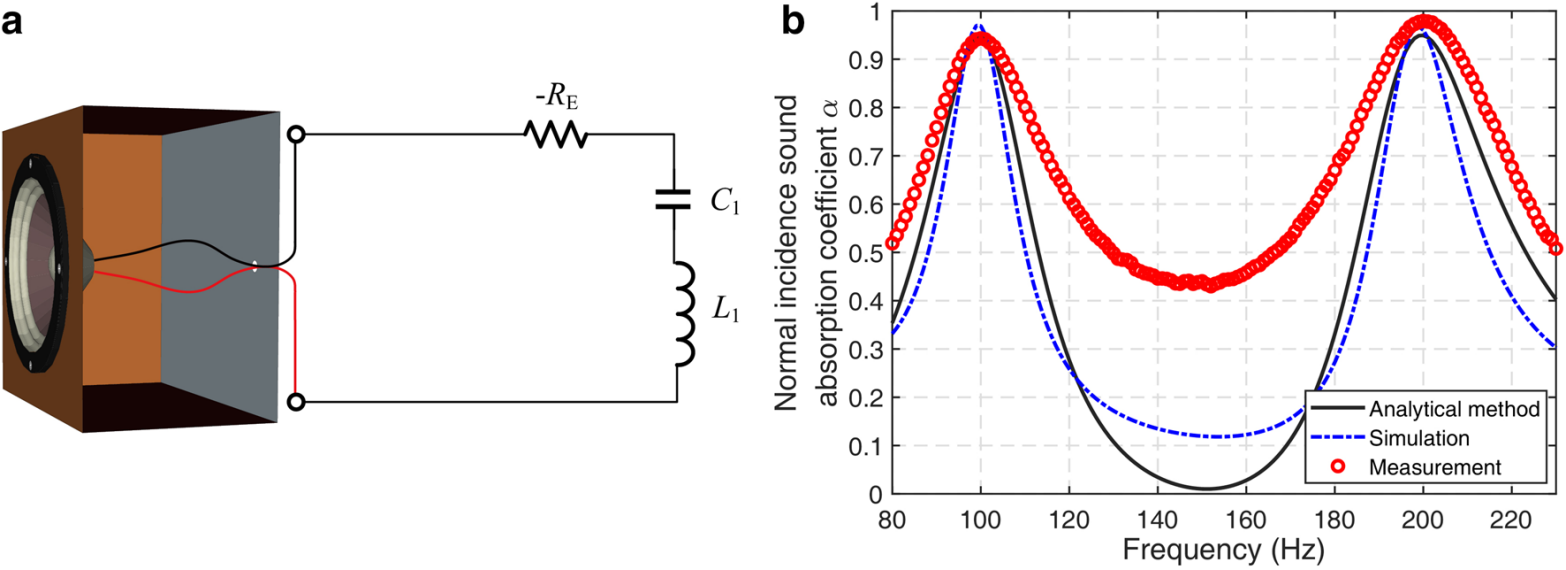
(**a**) The schematic of an element of the SL array for dual frequency sound absorption, (**b**) typical normal absorption coefficients of an element of the designed SL array [drawn with Microsoft Visio 2019 (https://itsc.nju.edu.cn/Visio/list.htm), MATLAB 2019a (https://itsc.nju.edu.cn/21628/list.htm) and Adobe Illustrator CC 2018 (https://itsc.nju.edu.cn/adobe/list.htm)].

Figure 1a shows an element of the SL array, which has a thickness of 11.8 cm and targets at 100 Hz and 200 Hz sound absorption. In the figure, − R_E_, *C*_1_ and *L*_1_ are negative shunt resistance, shunt capacitance and shunt inductance, respectively. The side length of the square front surface of the SL is 16.3 cm and the effective radius of the loudspeaker diaphragm is 5 cm. The Thiele-Small (TS) parameters^34,35^ of the loudspeaker driver and the electrical parameters of the shunt circuit are provided in the Supplementary Information. The normal absorption coefficient of each element in the array is calculated analytically (the details are listed in “The analytical method” section), simulated numerically (the details are listed in “The simulation method” section), and measured experimentally (the details are listed in “The measurement method” section), and the results are shown in Fig. 1b. The averaged values of the measured normal absorption coefficients agree well with that from the theory and simulations, and the values at 100 Hz and 200 Hz are 0.94 and 0.97, respectively.

**Figure 2:**
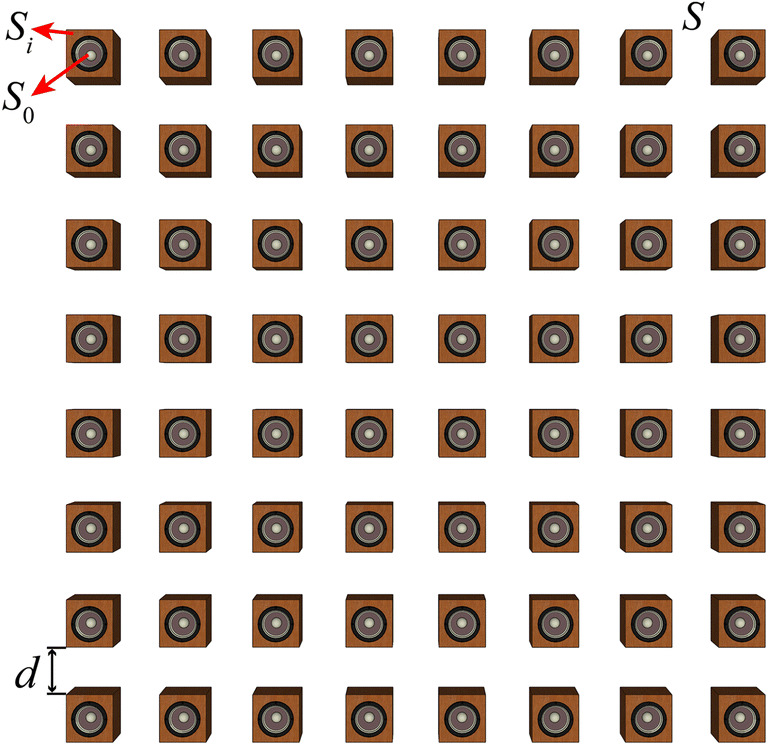
The schematic diagram of the SL array [drawn with Adobe Illustrator CC 2018 (https://itsc.nju.edu.cn/adobe/list.htm)].

Figure 2 shows the SL array made with 64 elements distributed evenly in an area of *S*, where *S*_0_ is the effective area of the speaker diaphragm, *S*_*i*_ is the area of the front surface of the *i*th element, and *d* is the interval between the elements. Define the area ratio *σ* as the ratio between the total effective area of the loudspeaker’s diaphragms to the area of the array *S*. The area ratio *σ* reaches the maximum value of 1 if *d* = 0 and *S*_*i*_ equals to *S*_0_, but for practical closed-box loudspeakers with a circular diaphragm, *σ* is less than π/4.

**Figure 3:**
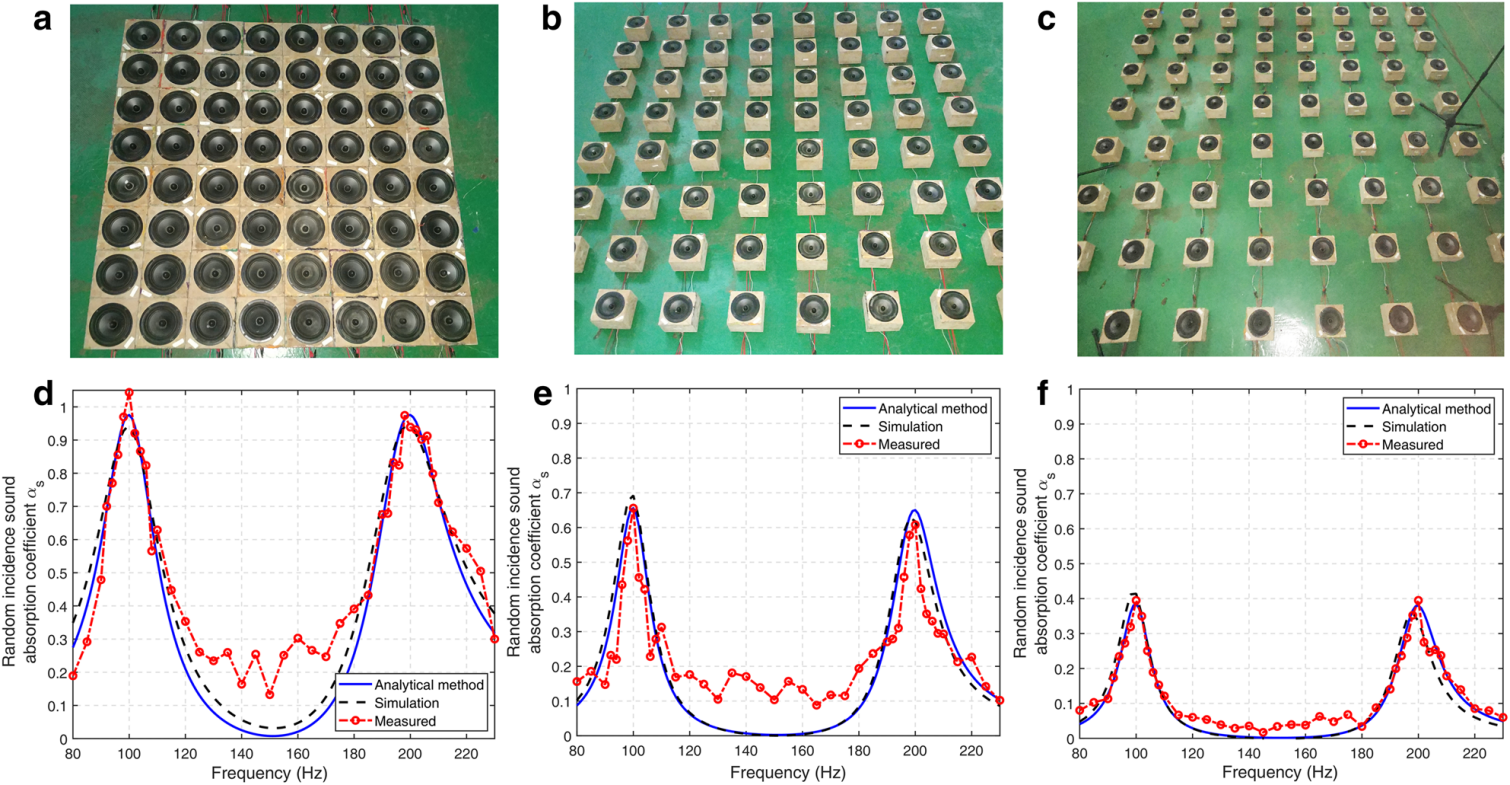
(**a**) The optimal layout of the SL array, (**b**) the SL array with the interval of 16.5 cm, (**c**) the SL array with the interval of 33 cm, (**d**) random incident absorption coefficient corresponding to configuration in (**a**), (**e**) random incident absorption coefficient corresponding to configuration in (**b**), (**f**) random incident absorption coefficient corresponding to configuration in (**c**) [drawn with MATLAB 2019a (https://itsc.nju.edu.cn/21628/list.htm) and Adobe Illustrator CC 2018 (https://itsc.nju.edu.cn/adobe/list.htm)].

Five layout patterns with different intervals of 33 cm, 25 cm, 16.5 cm, 8.3 cm and 0 cm were investigated and the random incident absorption coefficient was measured in the reverberation room. The optimal layout with the largest absorption coefficients at 100 Hz and 200 Hz is illustrated in Fig. 3a. In this layout, the interval between SLs is *d* = 0 m and the total area of the array is *S* = 1.74 m^2^. The random incident absorption coefficient of the array is 1.04 at 100 Hz and 0.93 at 200 Hz as shown in Fig. 3d. The main reason for the measured absorption coefficient in Fig. 3d to be greater than 1 is the extra sound absorption caused by the diffraction from the edges of the test sample to the incident sound wave^36^.

Figure 3b, c show two of the other four layout patterns with intervals of 16.5 cm and 33 cm, respectively. The areas of these two arrays are 6.10 m^2^ and 13.25 m^2^, so the area ratio *σ* are 0.082 and 0.038, respectively. The random incident absorption coefficients of the SL arrays with these two layouts are shown in Fig. 3e, f. The test results demonstrate that the proposed SL array has a good absorption performance while its thickness is only approximately 1/28 wavelength of 100 Hz.

## Discussions

**Figure 4:**
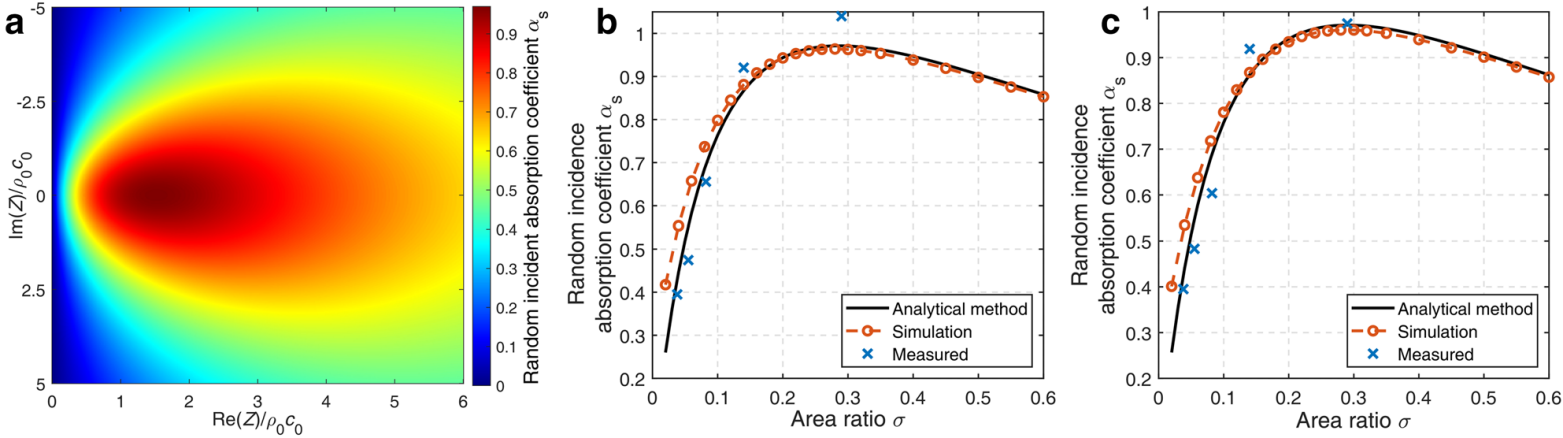
(**a**) The random incident absorption coefficient for the array of SLs with different acoustic impedance ratios, (**b**)–(**c**) random incident absorption efficient of the SL array with different area ratios at target frequencies (**b**) at 100 Hz (**c**) at 200 Hz [drawn with MATLAB 2019a (https://itsc.nju.edu.cn/21628/list.htm) and Adobe Illustrator CC 2018(https://itsc.nju.edu.cn/adobe/list.htm)].

The absorption coefficients of the SL array are determined by the properties of elements and their layout. The equivalent acoustic impedance *Z* of the array can be derived as

1 $$Z \approx \frac{{Z_{{{\text{SL}}}} }}{\sigma }$$

using the analytical method described in the “Methods” section, where *Z*_SL_ is the equivalent acoustic impedance of the SL. Figure 4a shows the random incident absorption coefficient of the SL array as a function of the equivalent acoustic impedance*.* When the real part of *Z* equals 1.51*ρ*_0_*c*_0_ and the imaginary part of *Z* is zero, where *ρ*_0_*c*_0_ is the characteristic acoustics impedance of air, the random incident absorption coefficient reaches a maximum value close to 0.97. As shown in Eq. (1), the equivalent acoustic impedance of the array is affected by the area ratio, so the maximum random incident absorption coefficient can be achieved by adjusting the area ratio *σ* when the real part of *Z*_SL_ is smaller than 1.51*ρ*_0_*c*_0_. The optimal area ratio *σ*_opt_ is

2 $$\sigma_{{{\text{opt}}}} { = }\frac{{{\text{Re}} \left( {Z_{{{\text{SL}}}} } \right)}}{{1.51\rho_{0} c_{0} }} = 0.66\frac{{{\text{Re}} \left( {Z_{{{\text{SL}}}} } \right)}}{{\rho_{0} c_{0} }},$$

where Re(*Z*_SL_) denotes the real part of *Z*_SL_.

Figure 4b,c shows the random incident absorption coefficients of the array with different area ratios at 100 Hz and 200 Hz, respectively, where the markers with “ × ” represent the measurement results. The theory and simulation results show that the random incident absorption coefficient increases first and then decreases with the area ratio *σ*, and the maximum absorption coefficient is 0.97 when the area ratio is 0.29 for both 100 Hz and 200 Hz. In the experiments, the maximal absorption coefficient at 100 Hz and 200 Hz appears when the interval is 0 cm as shown in Fig. 3a and the corresponding area ratio is 0.29. The optimal area ratio agrees well with the calculated value of 0.29 from Eq. (2).

In conclusion, the sound absorption coefficients of an array of shunt loudspeakers under random incidence were tested in a reverberation room and the results were analyzed with the parallel impedance method. With the optimized arrangement of the array elements, the proposed array achieves sound absorption coefficients of 1.04 and 0.93 at 100 Hz and 200 Hz, respectively, but with a thickness of only 1/28 of the wavelength of 100 Hz. It is demonstrated that an array of shunt loudspeakers can be designed as thin sound absorbers for low frequency noise control.

## Methods

### The analytical method

**Figure 5:**
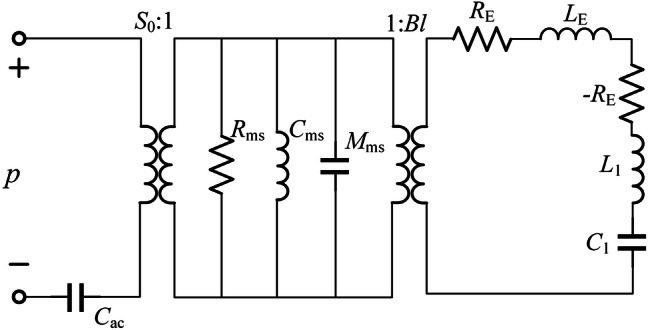
The equivalent circuit model of the designed SL prototype [drawn with Microsoft Visio 2019 (https://itsc.nju.edu.cn/Visio/list.htm)].

The equivalent acoustic impedance of the SL can be derived by using the equivalent circuit model^27^. Figure 5 shows the equivalent circuit model of a SL, where *p* is the incident pressure acting on the diagram, *Bl* is electromechanical coupling factor, *R*_E_ is the DC electrical resistance of the voice coil, *L*_E_ is the equivalent inductance of the voice coil, *R*_ms_ is the force resistance of the loudspeaker suspension system, *M*_ms_ is the mass of the driver cone, *C*_ms_ is the force compliance of the suspension system, and *S*_0_ is the effective area of the driver cone. *C*_ac_ is the equivalent acoustic capacitance of the back cavity with volume *V* and $C_{{{\text{ac}}}} = V/\rho_{0} c_{0}^{2}$. The values of all these parameters are given in the Supplementary Information. The acoustic impendence *Z*_SL_ at the diaphragm can be described by

3 $$Z_{{{\text{SL}}}} = \frac{{R_{{{\text{ms}}}} }}{{S_{0} }} + \frac{{j\omega M_{{{\text{ms}}}} }}{{S_{0} }} + \left( {\frac{1}{{j\omega C_{{{\text{ms}}}} S_{{0}}^{2} }} + \frac{1}{{j\omega C_{ac} }}} \right)S_{0} + \frac{{\left( {Bl} \right)^{2} }}{{S_{{0}} \left[ {j\omega \left( {L_{{\text{E}}} { + }L_{1} } \right) + {{1} \mathord{\left/ {\vphantom {{1} {j\omega C_{1} }}} \right. \kern-\nulldelimiterspace} {j\omega C_{1} }}} \right]}},$$

Assume *Z*_W_ is the acoustic impedance of the wooden plate with a thickness of 0.9 cm to make up the closed box, the equivalent acoustic impedance of each SL element can be obtained by

4 $$Z_{i} = \frac{{S_{i} }}{{\left( {\frac{{S_{0} }}{{Z_{{{\text{SL}}}} }} + \frac{{S_{i} - S_{0} }}{{Z_{{\text{W}}} }}} \right)}}.$$

The acoustic impedance of each space between SLs in the array can be obtained by^37^

5 $$Z_{{\text{s}}} = \frac{{\rho_{{0}} c_{0} }}{j\tan kh}$$

where *k* and *h* are the wave number and the thickness of the array respectively. The equivalent acoustic impedance of the whole SL array is

6 $$Z = \frac{S}{{\sum\nolimits_{{{\text{i}} = 1}}^{64} {(\frac{1}{{Z_{i} /S_{i} }}) + \frac{1}{{Z_{{\text{s}}} /(S - {64}S_{i} )}}} }}$$

When all SL elements are identical, using the relationship that *Z*_W_ >> *Z*_SL_ and *kh* << 1, Eq. (6) can be approximated as Eq. (1). And the random incident absorption coefficient *α*_s_ can be calculated by^38^

7 $$\alpha_{s} = \frac{{\int_{0}^{78^\circ } {\alpha \left( \theta \right)\sin \left( {2\theta } \right)d\theta } }}{{\int_{0}^{78^\circ } {\sin \left( {2\theta } \right)d\theta } }}$$

where the absorption coefficient of the SL array for the oblique incidence with an angle *θ* can be obtained by^37^

8 $$\alpha \left( \theta \right) = \frac{{4\rho_{0} c_{0} {\text{Re}} \left( Z \right)\cos \theta }}{{[\rho_{0} c_{0} + {\text{Re}} \left( Z \right)\cos \theta ]^{2} + [{\text{Im}} \left( Z \right)\cos \theta ]^{2} }}$$

**Figure 6:**
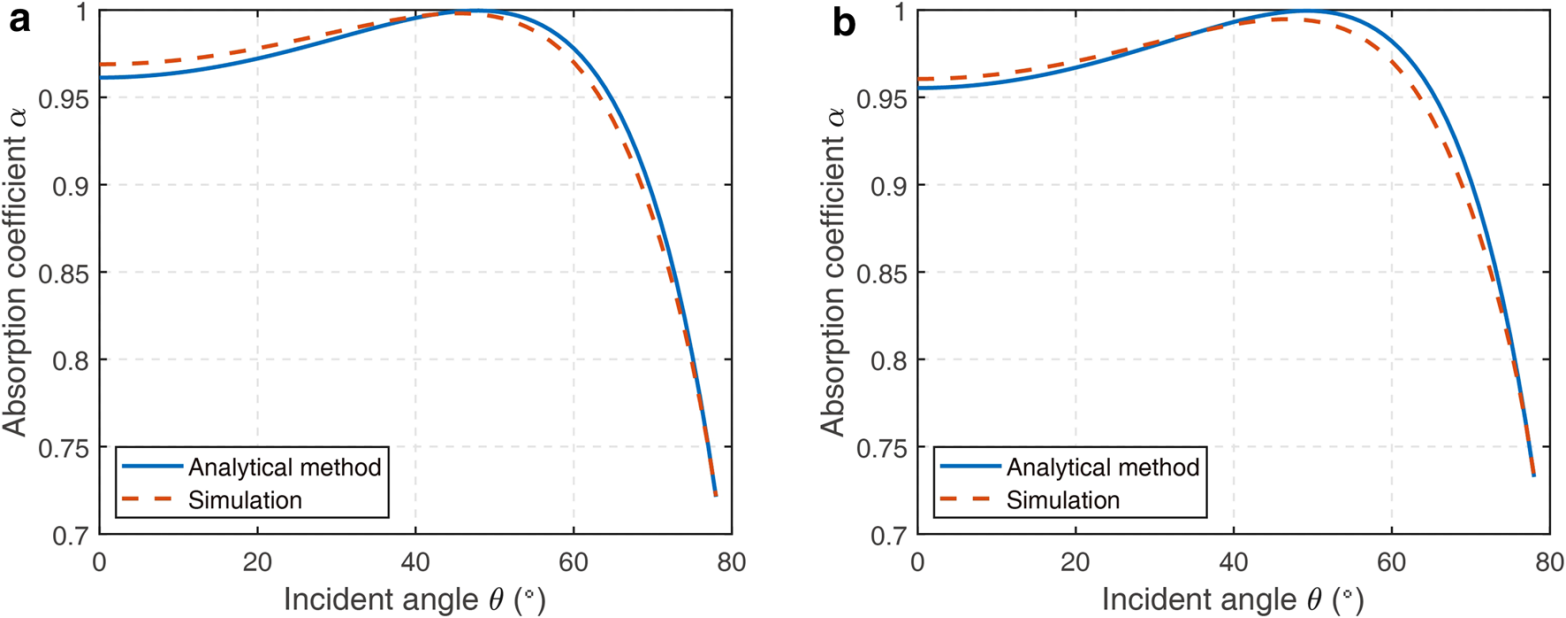
Oblique incident absorption coefficient with different incident angle when the space between the elements is *d* = 0 cm (**a**) at 100 Hz (**b**) at 200 Hz [drawn with MATLAB 2019a (https://itsc.nju.edu.cn/21628/list.htm)].

Figure 6 shows the calculated oblique incident absorption coefficient of the SL array at different incident angles at 100 Hz and 200 Hz when the space between the elements is *d* = 0 m. The simulation results are obtained with COMSOL Multiphysics. The absorption coefficients increase first and then decrease with the incident angle. The maximal oblique absorption coefficient at 100 Hz occurs at *θ* = 48° (Analytical method)/47° (Simulation), while the maximum at 200 Hz occurs at *θ* = 49° (Analytical method)/47° (Simulation).

### The simulation method

**Figure 7:**
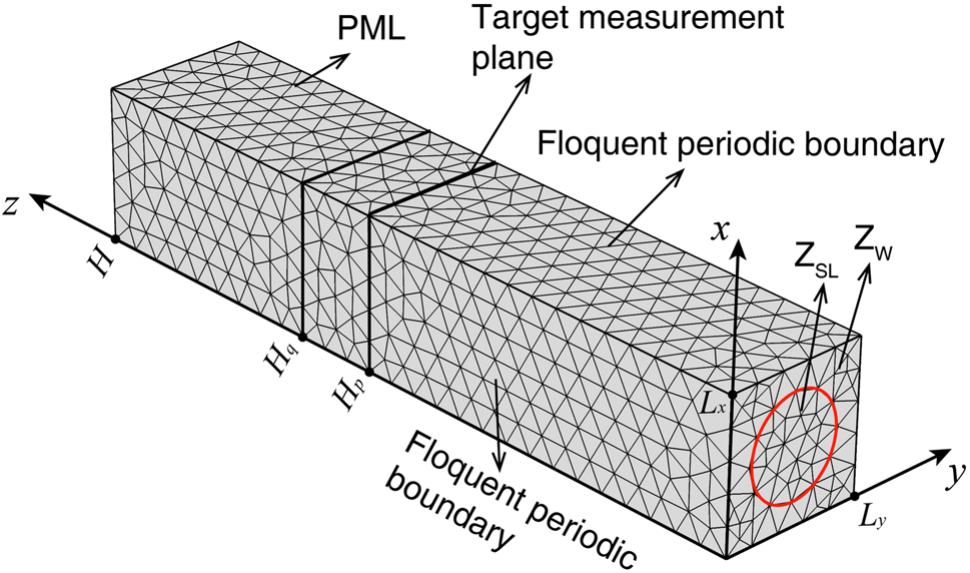
The finite element simulation model used in the research [drawn with COMSOL Multiphysics 5.4 (www.comsol.com) and Adobe Illustrator CC 2018 (https://itsc.nju.edu.cn/adobe/list.htm)].

In order to validate the analytical model, a numerical approach is adopted based on the Finite Element Method (FEM). The finite element simulation model established in COMSOL Multiphysics 5.3a is shown in Fig. 7. The surface at *z* = 0 is set as the impedance boundary, where the acoustic impedance in the middle circular region with radius of 5 cm is set as *Z*_SL_ and the rest area is set as a rigid wall. The region with 0 < *z* < *H*_*q*_ is set as the plane wave radiating sound field and the target measurement plane is *z* = *H*_*p*_. The region with *H*_*q*_ < *z* < *H* is set as a Perfectly Matched Layer (PML), which prevent the sound wave from reflecting when it propagates to the plane of *z* = *H*_q_. Thus, we can simulate a free field condition. The side surfaces with *x* = 0, *L*_*x*_ and *y* = 0, *L*_*y*_ are set as Floquent periodic boundaries, which makes the simulation model repeat periodically in both *x* and *y* directions. Thus, we can simulate an array with infinitely many shunt loudspeakers. The parameters *H*, *H*_*q*_, and *H*_*p*_ are set as 0.9 m, 0.6 m and 0.5 m respectively and *L*_*x*_, *L*_*y*_ are determined by different area ratio *σ*. The maximum element size is set as 3.43 cm and the geometric model is divided into 10,749 domain elements, 1,692 boundary elements, and 315 edge elements for numerical calculation.

When a background plane wave sound field with an incident angle *θ* is applied at the region of 0 < *z* < *H*_*q*_, the incident sound pressure *p*_inc_ and the reflected sound pressure *p*_scat_ on the target measurement plane at *z* = *H*_*p*_ are calculated. The sound absorption coefficient *α*(*θ*) could be obtained as

9 $$\alpha \left( \theta \right) = 1 - \left( {\frac{{p_{{{\text{inc}}}} }}{{p_{{{\text{scat}}}} }}} \right)^{2} .$$

The normal absorption coefficient can be calculated by Eq. (9) when the incident angle *θ* is 0°, and the random incident absorption coefficient is calculated by Eqs. (9) and  (7) when the incident angle *θ* is swept between 0° and 78° with a step of 1°.

### The measurement method

**Figure 8:**
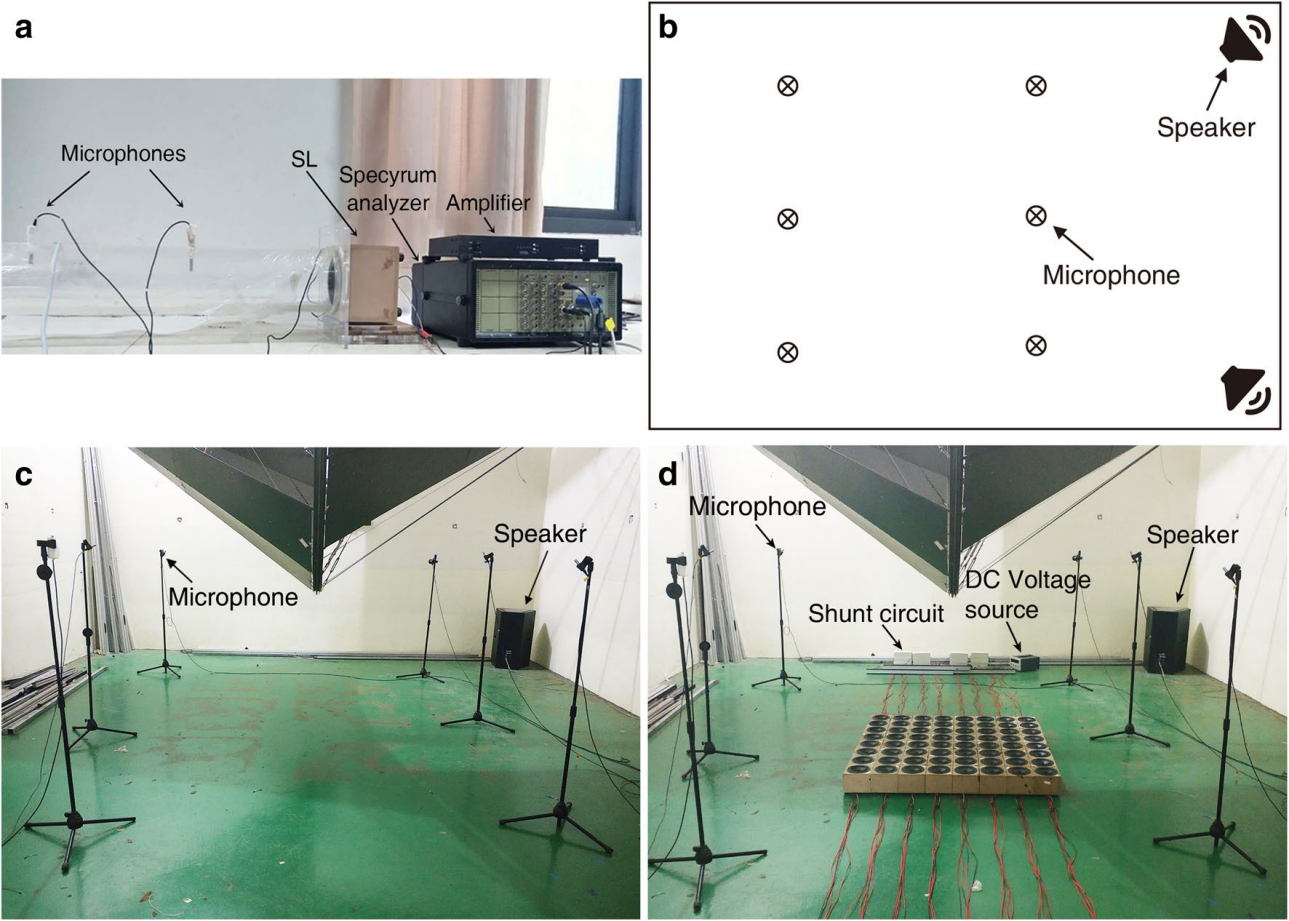
(**a**) Measurement apparatus for normal absorption coefficients, (**b**) the position of the microphones and the sound sources in the reverberation room, (**c**, **d**) the photo of the measurement setup without (**c**) and with (**d**) the shunt loudspeaker array in the reverberation room [drawn with Adobe Illustrator CC 2018 (https://itsc.nju.edu.cn/adobe/list.htm)].

The normal incident absorption coefficient was measured in an impedance tube according to ISO 10534-2^39^ with a B&K PULSE 3560D analyzer as shown in Fig. 8a. The sound source is fixed at the other end of the pipe and cannot be seen in the figure. The pipe is made of acrylic with a thickness of 15 mm, which can be considered as a rigid wall, because the acoustic characteristic impedance of acrylic is much larger than that of air. The diameter of the pipeline is 12 cm and the distance of the two microphones is 30 cm.

The measurement of the random incident absorption coefficient was conducted in the reverberation room of the Institute of Acoustics of Nanjing University and the sound source interruption method according to ISO 354:2003^40^ was adopted with a B&K PULSE 3560D analyzer. The volume of the reverberation chamber is 224.8 m^3^. The temperature and the relative humidity of air during the measurements were 25 °C and 72% respectively. Two *β*_3_-MU15 speakers manufactured by Elder Audio Manufacture Co., Ltd were used as the sound sources and placed at the corners of the reverberation room. Six microphones produced by Beijing AcousticSensing Technology Ltd were evenly placed in the reverberation room with a height of 1.2 m. The positions of the microphones and the sound sources are illustrated in Fig. 8b. The tonal sound ranging from 80 and 230 Hz with a step of 5 Hz was used in the measurements and the frequency step is reduced to 2 Hz between 90 and 110 Hz and between 190 and 210 Hz for better frequency resolution. Each measurement is repeated three times for averaging. A panoramic view of the measurement system without and with the SL array is shown in Fig. 8c,d, respectively.

## Supplementary information

Supplementary information

## Data Availability

All data generated or analyzed during this study are included in this published article (and its Supplementary Information files).
