# Supplementary material for: Dual frequency sound absorption with an array of shunt loudspeakers
Source: Sci Rep. 2020 Jul 2;10:10806. doi: 10.1038/s41598-020-67810-z (PMC7331664; doi:10.1038/s41598-020-67810-z)
Supplement: Supplementary file 1 — Supplementary information [file 41598_2020_67810_MOESM1_ESM.pdf]

Supplementary Information for

“Dual frequency sound absorption with an array of shunt loudspeakers”

Pengju Zhang[1], Chaonan Cong[2], Jiancheng Tao[1,*], Xiaojun Qiu[3]

*Correspondence to: jctao@nju.edu.cn

Details about the designed SL prototype

The designed SL prototype consists of a closed-box loudspeaker and a shunt circuit as shown in Fig. 1(a). The Thiele-Small (TS) parameters [S1-S2] of the loudspeaker driver were measured by using Klippel R&D System (Klippel GmbH Germany Company), and the values are shown in Supplementary Table S1 together with the electrical parameters of the shunt circuit. The external size of the closed-box backed the loudspeaker is 16.3 cm × 16.3 cm × 11.8 cm with the box wall thickness of 0.9 cm.

The circuit diagram of the negative impedance converter [S3] in the shunt circuit is shown in Supplementary Figure S1, where V_{cc+} and V_{cc-} are the supply voltages of the operational amplifier, U_i and U_o are the input and output voltages, R_1 and R_2 are two electrical resistances, and I is the input current. The ideal operational amplifier has zero input current and zero input offset voltage; therefore the input electrical impedance of the circuit in Supplementary Figure S1 is $-R_E R_1/R_2$. In the experiments, an operational amplifier OP07 was used, and both R_1 and R_2 were chosen as 300 Ω.

The shunt inductor L_1 itself has a certain parasitic resistance, so the negative resistance $-R_E$ needs to cancel both the DC resistance of the loudspeaker's voice coil and the parasitic resistance of the inductor L_1. This is the reason that the absolute value of the negative resistance presented in Supplementary Table S1 is greater than the DC resistance of the voice coil.

Supplementary Figures and Tables

Supplementary Table S1 | The parameters of the loudspeaker and the shunt circuit

Parameters	Notation	Value	Unit
Voice coil DC resistance	R_E	6.60	Ω
Voice coil inductance	L_E	0.84	mH
Electromechanical coupling factor	Bl	6.75	T·m
Moving mass	M_{ms}	8.40	g
Mechanical compliance	C_{ms}	0.65	mm/N
Mechanical resistance	R_{ms}	1.40	kg/s
Effective area	S_0	78.54	cm^2
Back cavity volume	V	2.1E-3	m^3
Shunt negative resistance	$-R_E$	–59.5	Ω
Shunt inductance	L_1	13.5	mH
Shunt capacitance	C_1	77	μF

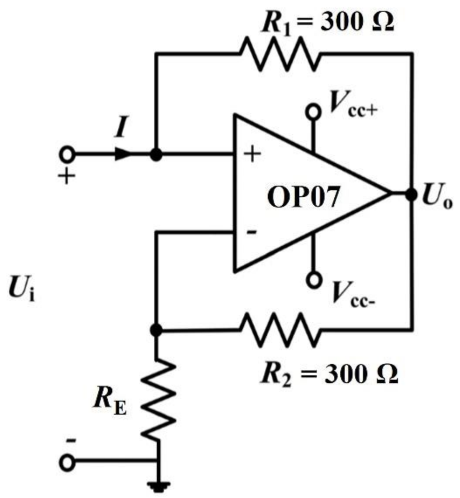

Supplementary Figure S1 | The circuit diagram of the negative impedance converter

References

[S1]. Thiele, N. Loudspeakers in vented boxes: Part 2. *J. Audio. Eng. Soc.* **19(5)**, 471-483 (1971).

[S2]. Small, R. H. Direct radiator loudspeaker system analysis. *J. Audio. Eng. Soc.* **20(5)**, 383-395 (1972).

[S3]. Černík, M. & Mokrý, P. Sound reflection in an acoustic impedance tube terminated with a loudspeaker shunted by a negative impedance converter. *Smart Mater. Struct.* **21**(11), 115016 (2012).
